# The usefulness of CorvisST Tonometry and the Ocular Response Analyzer to assess the progression of glaucoma

**DOI:** 10.1038/srep40798

**Published:** 2017-01-17

**Authors:** Masato Matsuura, Kazunori Hirasawa, Hiroshi Murata, Shunsuke Nakakura, Yoshiaki Kiuchi, Ryo Asaoka

**Affiliations:** 1 Department of Ophthalmology, University of Tokyo Graduate School of Medicine, Tokyo, Japan; 2 Orthoptics and Visual Science, Department of Rehabilitation, School of Allied Health Sciences, Kitasato University, Kanagawa, Japan; 3 Department of Ophthalmology, Saneikai Tsukazaki Hospital, Himeji, Japan; 4 Department of Ophthalmology and Visual Science, Hiroshima University, Hiroshima, Japan

## Abstract

Corneal Visualization Scheimpflug Technology (CST) and Ocular Response Analyzer (ORA) measurements were carried out in 105 eyes of 69 patients with primary open-angle glaucoma. All patients had axial length (AL), central corneal thickness (CCT), intraocular pressure (IOP) with Goldmann applanation tonometry (GAT) and eight visual fields (VF)s with the Humphrey Field Analyzer. VF progression was summarized using a time trend analysis of mean total deviation (mTD) and the association between mTD progression rate and a number of ocular parameters (including CST and ORA measurements) was assessed using mixed linear regression analysis. The optimal model of VF progression selected based on the corrected Akaike Information Criteria (AICc) included ORA’s corneal hysteresis (CH) parameter as well as a number of CST measurements: mTD progression rate = 1.2–0.070 * mean GAT + 0.090 * CH–1.5 * highest concavity deformation amplitude with CST + 9.4 * A1 deformation amplitude with CST–0.05 * A2 length with CST (AICc = 125.8). Eyes with corneas that experience deep indentation at the maximum deformation, shallow indentation at the first applanation and wide indentation at the second applanation in the CST measurement are more likely to experience faster rates of VF progression.

Glaucoma is the second leading cause of blindness worldwide, affecting approximately 60 million peoples^1^. The disease causes irreversible visual field (VF) damage so it is very important to predict its progression and make appropriate interventions as soon as possible. The principal target of glaucoma treatments is to reduce and control intraocular pressure (IOP), which has been shown to reduce VF progression by numerous clinical trials and research studies^2^,^3^,^4^,^5^,^6^,^7^,^8^,^9^,^10^. Tonometry measurements of IOP can be greatly influenced by structural properties of the eye; in particular, IOP measured with Goldmann applanation tonometry (GAT) has been shown to be affected by central corneal thickness (CCT)^11^,^12^,^13^,^14^,^15^,^16^,^17^,^18^,^19^,^20^,^21^,^22^,^23^. Thus, CCT should be considered when interpreting GAT-measured IOP and making clinical decisions. Furthermore, studies have suggested that CCT is associated with the progression of glaucoma^4^,^24^. Other biomechanical properties of the cornea have also been shown to affect the progression of glaucoma. In particular, corneal hysteresis (CH) and corneal resistance factor (CRF), measured with the Ocular Response Analyzer (ORA, Reichert Ophthalmic Instruments, Depew, NY, USA), have been reported to impinge on progression^25^,^26^.

The Corneal Visualization Scheimpflug Technology instrument (Corvis ST tonometry: CST; Oculus, Wetzlar, Germany) is a new device, integrated with an ultra-high-speed Scheimpflug camera, to quantitatively measure biomechanical properties of the cornea during the application of a rapid air-puff^27^. As a result, very detailed corneal movement during the air puff application can be observed, such as velocity of corneal deformation at the first and second applanations and the maximum depth of corneal deformation (Fig. 1). Although CST and ORA both measure the biomechanical properties of the cornea, their mechanisms are completely different, and the relationship between CST-measured corneal parameters and the progression of glaucomatous VF damage has not been reported in detail. Jung *et al*. have reported the relationship between maximum depth of corneal deformation against peripapillary atrophy area and the disc tilt ratio in patients with glaucoma^28^, however more detailed investigation has not been reported, such as the relationship between visual field progression rate and various (twelve) CST parameters, including maximum depth of corneal deformation.

Therefore, the purpose of the current study is to investigate the effect of ORA- and CST-measured parameters on the progression of glaucomatous VF damage in patients with primary open angle glaucoma (POAG).

## Method

The study was approved by Research Ethics Committee of the Graduated School of Medicine and Faculty of Medicine at The University of Tokyo. Written informed consent was given by patients for their information to be stored in the hospital database and used for research. This study was performed according to the tenets of the Declaration of Helsinki.

### Subjects

One hundred and five eyes of 69 POAG patients (36 males and 33 females) were included in this study. POAG was defined as (1) presence of typical glaucomatous changes in the optic nerve head such as a rim notch with a rim width ≤0.1 disc diameters or a vertical cup-to-disc ratio of >0.7 and/or a retinal nerve fiber layer defect with its edge at the optic nerve head margin greater than a major retinal vessel, diverging in an arcuate or wedge shape; and (2) gonioscopically wide open angles of grade 3 or 4 based on the Shaffer classification. All patients had at least 9 VFs measured with the Humphrey Field Analyzer II (HFA, Carl Zeiss Meditec Inc., Dublin, CA, USA), with the SITA standard 24-2 or 30-2 program. Reliable VFs were defined as Fixation loss (FL) rate <20% and False positive (FP) rate <15% following the criteria used in the HFA software; false negative (FN) was not used as an exclusion criterion. Reliable VFs were identified and eyes with at least eight reliable VFs were investigated, excluding the first VF measurement. We chose a minimum of eight VFs because it has recently been reported that this number is needed to precisely analyze VF progression^29^,^30^,^31^,^32^,^33^. Eyes that experienced any surgical procedure, including trabeculectomy and cataract surgery, during or prior to this VF series period were excluded. Inclusion criteria were no abnormal eye-related findings except for OAG on biomicroscopy, gonioscopy and funduscopy. Eyes with a history of other ocular disease, such as age-related macular degeneration were also excluded. Eyes with significant cataract which affects VF were carefully excluded. Only subjects aged ≧20 years old were included and eyes with IOP > 25 mmHg or contact lens wearers were excluded. If both eyes satisfied the inclusion criteria, then both were included in the study. Axial length (AL) and CCT were also measured in all patients using the IOL Master, ver. 5.02 (Carl Zeiss Meditec Inc., Dublin, CA, USA) and CST, respectively. CST was performed after the final (8th) VF measurement.

### ORA measurements

ORA records two applanation pressure measurements, prior to and following indentation by a rapid jet of air. Due to its viscoelastic property, the cornea resists the air puff, resulting in delays in the inward and outward applanation events, which causes a measureable difference in air puff values. This difference is called CH, while CRF represents the resistance of cornea^34^.

Both ORA and CST were carried out three times (on the same day) prior to the GAT-IOP measurement and within 180 days from the eight VF measurement. The average value of the three measurements was used in the analyses. The order of ORA and CST measurements was decided randomly. All data were of sufficient quality, as guaranteed by analyzing only eyes with a quality index >7.5.

### Corvis ST tonometer measurements

The principles of CST are described in detail elsewhere^27^. In short, the instrument’s camera records a sequence of images that capture corneal deformation following a rapid air puff. The device is capable of capturing 4,330 images per second that are analyzed to quantify CCT, deformation amplitude, applanation length and corneal velocity. Each measurement is further distinguished as follows: ‘A1/A2 time’ is the length of time from the initiation of the air puff to the first (cornea moves inwards) or second applanation (cornea moves outwards); ‘A1/2 length’ is the length of the flattened cornea at the first or second applanation; ‘A1/2 velocity’ is the velocity of the movement of cornea during the first or second applanation; ‘A1/2 deformation amplitude’ is the movement of the corneal apex of the flattened cornea at the first or second applanation; ‘peak distance’ is the distance between the two surrounding peaks of the cornea at the highest concavity; ‘highest concavity deformation amplitude’ is the magnitude of movement of the corneal apex from before deformation to its highest concavity: ‘highest concavity time’ is the length of the time taken to reach highest concavity from pre-deformation of the cornea; ‘radius’ is the central curvature radius at the point of highest concavity.

CST (software version; 1.2r1092) was performed three separate times, on the same day, with at least a one minute interval between each repeat measurement of ORA and CST. Averages of CST parameters were calculated for the three repeated tests. All CST measurements were considered reliable according to the “OK” quality index displayed on the device monitor.

### Other measurements

The mean and standard deviation (SD) of all GAT-IOP measurements during the follow up period were calculated.

### VF data

The mean total deviation (mTD) of the 52 test points used in the 24-2 HFA VF test pattern was calculated, for both of 24-2 and 30-2 HFA VFs. The progression rate of mTD was calculated using the eight VFs collected from each eye, similarly to the MD trend analysis employed in the HFA.

### Statistical analysis

The relationship between ORA (CH and CRF), CST parameters (A 1/2 time, A 1/2 length, A 1/2 velocity, A 1/2 deformation amplitude, highest deformation amplitude, highest concavity time, peak distance, and radius) and other ocular/systemic parameters (age, mean GAT, SD of GAT, CCT, AL, and mTD in the initial VF) against mTD progression rate was investigated using a linear mixed model with patient as a random effect (because one or two eyes of a patient were included). The optimal linear mixed model (model_basic_) to describe mTD progression rate using ocular/systemic parameters was selected according to the second order bias corrected Akaike Information Criterion (AICc) index. Two further models were selected adding only ORA parameters and also both ORA and CST parameters (model_ORA_ and model_ORA_CST_, respectively). The AICc is the corrected form of the common statistical measure of AIC. AICc gives an accurate estimation even when the sample size is small^35^. In a multivariate regression model, degrees of freedom decreases as the number of variables increases, hence it is recommended to use model selection methods to improve the model fit by removing redundant variables^36^,^37^. Any magnitude of reduction in AICc suggests an improvement of the model, and the probability that one particular model is the model that minimizes ‘information loss’ can be calculated; when there are *n* candidate models and the AICc values of those models are AIC_1_, AIC_2_, AIC_3_, …, AIC_*n*_. If AIC_min_ is the minimum of these values then exp((AIC_min_ − AIC_*i*_)/2) describes the relative probability that the *i*th model minimizes the information loss (i.e. is the ‘optimal model’)^38^. Relative probabilities were calculated among all candidate models. All statistical analyses were performed using the statistical programming language ‘R’ (R version 3.2.3; The foundation for Statistical Computing, Vienna, Austria).

## Results

Characteristics of the study subjects are summarized in Table 1. The mean ± standard deviation (SD) [range] age was 63.2 ± 9.7 [43 to 85], 36 patients were male and 33 patients were female. Eight VFs were measured over an average period of 2412.2 ± 868.9 [630 to 6881] days. GAT was conducted 29.0 ± 7.1 [18 to 69] times during the follow up period (between the initial VF and the eighth VF). Mean GAT-IOP was 13.5 ± 2.2 [8.9 to 20.2] mmHg with an SD value of 1.5 ± 0.47 [0.79 to 3.6].

Summary statistics of CST and CH measurements are shown in Table 2. The modelled relationships between mTD progression rate and mean GAT, SD of GAT, ORA parameters and CST parameters are shown in Table 3. A significant relationship was observed for age and CH (p = 0.032 and 0.049, respectively, linear mixed model).

As a result of model (parameter) selection, the optimum equation for model_basic_ was: mTD progression rate = 0.32 (intercept) −0.0094 * age (AICc = 132.3); thus all other variables (mean GAT, SD of GAT, CCT, AL and mTD in the initial VF) were not deemed to improve the model. The equation for model_ORA_ was: mTD progression rate = −0.35 −0.0083 * age + 0.065 * CH (AICc = 131.4). The equation for model_ORA_CST_ was: mTD progression rate = 1.2–0.070 * mean GAT + 0.090 * CH + −1.5 * highest deformation amplitude + 9.4 * A1 deformation amplitude −0.05 * A2 length (AICc = 125.8). The probability that model_ORA_ minimizes information loss compared to model_basic_ was 36.2%. The probability that model_ORA_CST_ minimizes information loss compared to model_ORA_ and model_basic_ was 93.9% and 96.1%, respectively.

## Discussion

In the current study CST and ORA measurements were carried out in 105 eyes of 69 patients with POAG. In models describing the progression rate of mTD, the inclusion of CH and CST parameters resulted in the most favorable model. This model included mean GAT-IOP (higher IOP leads to faster progression) and CH (lower CH indicates faster progression) agreeing with previous studies^4^,^25^,^26^,^39^,^40^,^41^, as well as highest concavity deformation amplitude (the higher the amplitude, the faster progression), A1 deformation amplitude (lower amplitude suggests faster progression), A2 length (larger length leads to faster progression).

Interestingly, model_basic_ did not include mean GAT-IOP, despite its undoubted influence on VF progression^3^,^42^,^43^,^44^. However, this does not deny the importance of IOP control in the management of glaucoma. The current study analyzed clinical data from a real world setting where IOP reduction interventions would be intensified if VF progression was identified. This result aligns with our recent findings from a multi-central study in which the real world clinical data was analyzed and a relationship between mean IOP and the progression of glaucoma was not found^45^. In the same study, however, the SD of IOP measurements was related to the progression of glaucoma^45^. These findings are probably because patients’ VF progression is effectively controlled by reducing mean IOP, but SD of IOP measurements is less considered in the clinical treatment decision.

Only the age variable was selected in model_basic_. Age has been reported elsewhere to be an independent risk factor of the progression of glaucoma^4^,^39^,^40^,^41^. Surprisingly, age was not included in model_ORA_CST_, however, this model did include CH, which may act as a proxy for age since CH decreases as age increases^46^. Further, CST parameters are also included in model_ORA_CST_ and some CST parameters are also related to age; in particular, highest deformation amplitude increases as age increases. Thus, the current results may suggest that the accelerating effect of age on VF progression may actually be driven a change in CH and/or highest deformation amplitude. This is clinically very important because an eye with a low CH and a large highest deformation amplitude has a greater risk of progression at any age.

CCT has also been reported to be associated with the progression of glaucoma^4^,^11^,^13^,^24^,^47^,^48^, however, CCT was not selected in any of our models in the current study. Recent studies have reported that CH is a stronger risk factor for the progression of glaucoma than CCT^26^, and indeed, in the current study, CH was included in both model_ORA_ and model_ORA_CST_. Baseline VF damage may^4^,^39^,^49^ or may not^40^,^50^,^51^ be related to faster VF progression; however, in the current study, mTD in the initial VF was not included in any of the optimal models.

Interestingly, the AICc of model_ORA_CST_ was significantly smaller than the AICc of model_ORA_ (according to their AIC values, model_ORA_CST_ is deemed to be the better model with a probability of 93.9%). Thus, it may be advantageous to use CST, in addition to ORA, to better interpret VF progression in glaucoma patients. In particular, model_ORA_CST_ suggests that eyes with corneas experiencing a deep indentation following the CST air-puff are likely to progress at a faster rate. The hysteresis of a viscoelastic material is defined as the amount of energy absorption during the ‘loading/unloading’ stress/strain cycle and the magnitude of the energy absorption can be calculated as the area surrounded by the loading and unloading curves^52^, which is thought to reflect less compliance of the lamina cribrosa and thus provide further information about glaucoma risk^25^,^53^. A deeper highest deformation amplitude indicates the change of shape of the loading/unloading curves. Its effect on the progression of glaucoma has not been examined in detail so a further study should be carried out to shed light on this finding. Further, a wide applanated area at the second applanation (A2 length) may be related to a deeper indentation at the maximum deformation. Smaller movements of the corneal apex at the first applanation were related to faster progression in the current study. A smaller movement of the corneal apex at the first applanation may indicate that the absorption of the projected energy is finished at the phase of the first applanation. An eye exhibiting this ‘poor’ absorption of energy could be indented more deeply at the highest deformation. Again, a further study is needed to investigate these findings in more detail.

A limitation of the current study is that we could not control for the effect of anti-glaucomatous eye drops on corneal biomechanical properties^54^,^55^,^56^,^57^. As all patients were recruited from real world glaucoma clinics this could not be avoided, but it could have a non-negligible effect on the study findings. In addition, ORA and CST measured values may change over a long-term follow-up. As there is no study which investigated the variability of CST parameters in long-term follow-up, it should be investigated in a future study. Also, it is the mTD (or mean deviation) slope trend analysis most frequently used at the clinical settings, and hence we used this method to evaluate VF progression. However it is also true mTD slope analysis may miss focal progression. There is no gold standard method to evaluate the focal progression, but it may be the best way to divide VF into small clusters and evaluate the influence of CST parameters in each sector, which should be carried out in a following study. Also, it would be needed to further confirm the current result using an independent population.

In conclusion, it is advantageous to carry out CST tonometry in addition to ORA when assessing the progression of glaucomatous VF change. Careful glaucoma management is required in eyes with any of the following characteristics: a low CH, a large highest concavity deformation amplitude, a large A2 length, or, a small A1 deformation amplitude since these eyes are at greatest risk of VF progression.

## Additional Information

**How to cite this article**: Matsuura, M. *et al*. The usefulness of CorvisST Tonometry and the Ocular Response Analyzer to assess the progression of glaucoma. *Sci. Rep.* **7**, 40798; doi: 10.1038/srep40798 (2017).

**Publisher's note:** Springer Nature remains neutral with regard to jurisdictional claims in published maps and institutional affiliations.

## Figures and Tables

**Figure 1:**
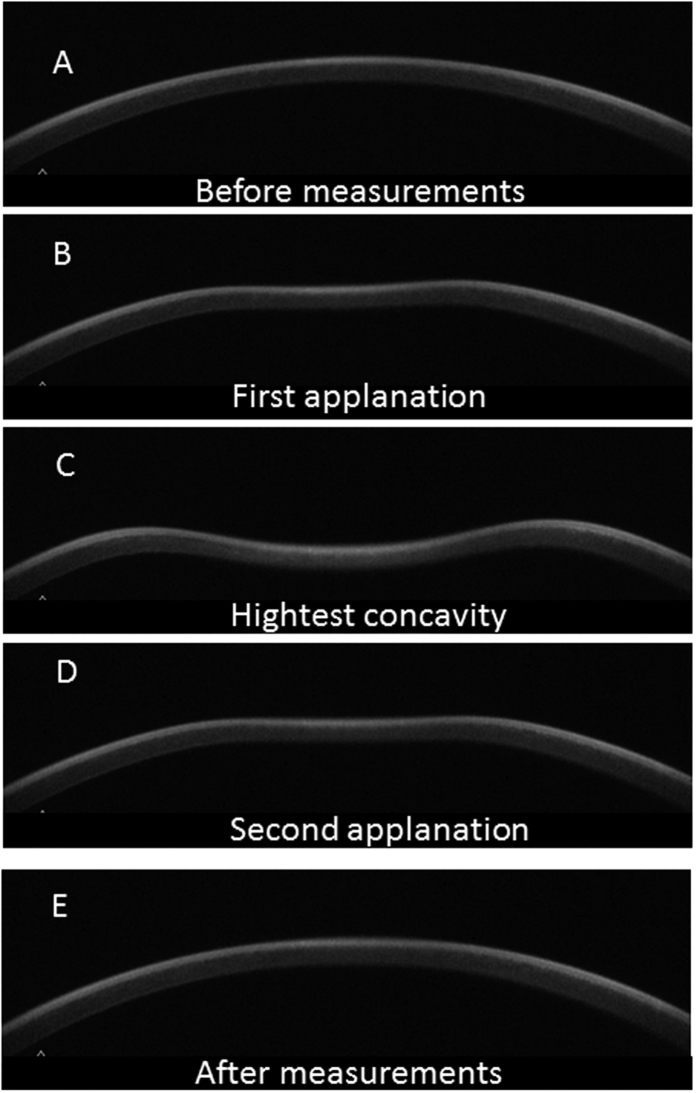
Corneal movement during the CST measurement. In the CST tonometry measurement, a rapid air puff is applied to cornea and cornea moves inward whereas ORA measures air jet pressure at the events of first and second applanations. The figures show the corneal shape in each phase: (**A**) prior to air puff applanation, (**B**) first applanation, (**C**) highest concavity, (**D**) second applanation, and (**E**) posterior to air puff applanation. CST: Corvis ST tonometry, ORA: Ocular Response Analyzer.

**Table 1:** Subject demographics.

Variables	Value
age, (mean ± SD) [range], years old	63.2 ± 9.7 [43 to 85]
Male/Female	36/33
Right/Left	50/55
GAT, (mean ± SD) [range], mmHg	13.5 ± 2.2 [8.9 to 20.2]
AL, (mean ± SD) [range], mm	25.1 ± 1.6 [22.3 to 29.2]
CCT, (mean ± SD) [range], μm	530.9 ± 35.6 [458.3 to 624.3]
mTD, (mean ± SD) [range], dB	−6.8 ± 6.5 [−27.0 to 3.9]

sd: standard deviation, GAT: intraocular pressure measured with Goldmann tonometry, AL: axial length, CCT: central corneal thickness, mTD: mean of total deviation values.

**Table 2:** CST and ORA parameters.

	Parameter	Value (mean ± sd) [range]
ORA	CH (mmHg)	9.2 ± 1.1 [6.5 to 11.8]
	CRF (mmHg)	8.4 ± 1.4 [4.9 to 13.0]
CST	A1 time (ms)	7.2 ± 0.3 [6.5 to 8.4]
	A1 length (mm)	1.7 ± 0.072 [1.4 to 1.8]
	A1 velocity (m/s)	0.16 ± 0.014 [0.10 to 0.20]
	A1 deformation amplitude (mm)	0.12 ± 0.0083 [0.11 to 0.16]
	A2 time (ms)	21.9 ± 0.46 [20.9 to 23.2]
	A2 length (mm)	1.7 ± 0.23 [0.83 to 2.2]
	A2 velocity (m/s)	−0.39 ± 0.079 [−0.16 to −0.63]
	A2 deformation amplitude (mm)	0.41 ± 0.072 [0.25 to 0.57]
	highest deformation amplitude (mm)	1.1 ± 0.11 [0.82 to 1.3]
	highest concavity time (ms)	16.9 ± 0.57 [15.4 to 18.4]
	Peak distance (mm)	3.4 ± 0.88 [2.1 to 5.5]
	Radius (mm)	7.5 ± 0.85 [6.2 to 10.3]

sd: standard deviation, CCT: central corneal thickness, CH: corneal hysteresis, CRF: corneal resistant factor.

**Table 3:** The relationship between CST/ORA parameters and various ocular parametetrs, and visual field progression rate.

	Coefficient	Standard error	p value	AICc
age (years old)	**0**.**0094**	**0**.**0043**	**0**.**032**	**132**.**3**
mean GAT (mmHG)	−0.017	0.019	0.38	136.2
SD of GAT (mmHG)	−0.076	0.092	0.41	136.3
CCT (mm)	0.00070	0.00120	0.52	136.5
axial length (mm)	0.019	0.026	0.47	136.5
mTD in the initial VF	0.0057	0.0057	0.40	136.2
A1 time (ms)	0.21	0.15	0.16	134.9
A1 length (mm)	−0.20	0.60	0.74	136.9
A1 velocity (m/s)	−2.7	3.1	0.37	136.3
A1 deformation amplitude (mm)	6.7	5.2	0.20	135.1
A2 time (ms)	−0.026	0.094	0.78	136.9
A2 length (mm)	−0.18	0.19	0.33	136.1
A2 velocity (m/s)	0.72	0.55	0.20	135.1
A2 deformation amplitude (mm)	−0.11	0.60	0.86	136.9
highest deformation amplitude (mm)	−0.59	0.39	0.13	134.7
highest concavity time (ms)	0.0019	0.075	0.98	137.0
Peak distance (mm)	−0.053	0.051	0.30	135.4
Radius (mm)	0.072	0.050	0.15	134.8
CH (mmHg)	**0**.**076**	**0**.**038**	**0**.**049**	**132**.**8**
CRF (mmHg)	0.045	0.030	0.14	134.6

Bold characters represent p < 0.05.

CST: Corvis ST tonometry, ORA: ocular response analyzer.
